# Psycho-physiological assessment of a prosthetic hand sensory feedback system based on an auditory display: a preliminary study

**DOI:** 10.1186/1743-0003-9-33

**Published:** 2012-06-09

**Authors:** Jose Gonzalez, Hirokazu Soma, Masashi Sekine, Wenwei Yu

**Affiliations:** 1 Medical System Engineering Department, Chiba University, Chiba, Japan; 2 Research Center for frontier Medical Engineering, Chiba University, Chiba, Japan

## Abstract

**Background:**

Prosthetic hand users have to rely extensively on visual feedback, which seems to lead to a high conscious burden for the users, in order to manipulate their prosthetic devices. Indirect methods (electro-cutaneous, vibrotactile, auditory cues) have been used to convey information from the artificial limb to the amputee, but the usability and advantages of these feedback methods were explored mainly by looking at the performance results, not taking into account measurements of the user’s mental effort, attention, and emotions. The main objective of this study was to explore the feasibility of using psycho-physiological measurements to assess cognitive effort when manipulating a robot hand with and without the usage of a sensory substitution system based on auditory feedback, and how these psycho-physiological recordings relate to temporal and grasping performance in a static setting.

**Methods:**

10 male subjects (26+/-years old), participated in this study and were asked to come for 2 consecutive days. On the first day the experiment objective, tasks, and experiment setting was explained. Then, they completed a 30 minutes guided training. On the second day each subject was tested in 3 different modalities: Auditory Feedback only control (AF), Visual Feedback only control (VF), and Audiovisual Feedback control (AVF). For each modality they were asked to perform 10 trials. At the end of each test, the subject had to answer the NASA TLX questionnaire. Also, during the test the subject’s EEG, ECG, electro-dermal activity (EDA), and respiration rate were measured.

**Results:**

The results show that a higher mental effort is needed when the subjects rely only on their vision, and that this effort seems to be reduced when auditory feedback is added to the human-machine interaction (multimodal feedback). Furthermore, better temporal performance and better grasping performance was obtained in the audiovisual modality.

**Conclusions:**

The performance improvements when using auditory cues, along with vision (multimodal feedback), can be attributed to a reduced attentional demand during the task, which can be attributed to a visual “pop-out” or enhance effect. Also, the NASA TLX, the EEG’s Alpha and Beta band, and the Heart Rate could be used to further evaluate sensory feedback systems in prosthetic applications.

## Background

It is well known that upper limb amputees have to rely extensively on visual feedback in order to monitor and manipulate successfully their prosthetic device. This situation seems to lead to a high conscious burden for the users, which generates fatigue and frustration [1,2]. This lack of sensory feedback is a major drawback that many researchers are trying to cope with by using indirect methods to convey information from the artificial limb to the amputee, such as electro-cutaneous stimulation [3-8], vibrotactile stimulation [8-12], force stimulation [13-18], or auditory cues [19-22]. Although the results obtained are very positive, the usability and advantages of these feedback methods were explored mainly by looking at the performance results, which do not take into account measurements of the user’s mental effort, attention, and emotions. As a different approach, A. Hernandez et al., in [3], explored the effect of electro-tactile feedback on amputees’ cerebral cortex using fMRI data, showing that the motor-sensory areas of the lost limb in a subject’s brain were activated when the subject grabbed an object with a prosthetic hand while looking at the action and feeling the electrical stimulation. This result gives good insight of the brain plasticity, but still the authors didn’t address the mental load due to the multimodal information display. It has been claimed or assumed in literature that, by adding a feedback loop to the prosthetic control, the users will improve his performance and awareness of the robot hand, reducing their consciousness burden [1-3,6-13,17]. However, to the best of our knowledge, there haven’t been studies supporting this claim or assumption directly. The evaluation methods used in these studies had focused only on performance results and informal reports. Therefore, it is possible that by using an extra feedback loop, the performance will be improved, but at the expense of a higher mental effort, driving the amputee to fatigue faster. Therefore, it is reasonable to question how does the presentation of multimodal information affect the robot hand user? Is the mental workload increasing or decreasing?

In order to measure mental workload, different methods have been used in the area of human machine interaction and psychology. The most common method used is self-assessment questionnaires, for example, the NASA TLX questionnaire [23,24]. This method has proven to be very reliable for different applications, but there are problems with validity and corroboration since subjects can answer differently from what they are really feeling or they might be confused by the questions and not answer correctly. Another disadvantage is that continuous monitoring cannot be accomplished. Therefore, to validate and corroborate these self-report measurements, psycho-physiological measurements have been widely used. By measuring changes in the autonomic nervous system (ANS) and the central nervous system (CNS), it is possible to directly and continuously monitor changes in cognitive activity and emotions of a person when carrying out different tasks [25]. This method has been used to assess mental effort [26-29], to measure user experiences in entertainment technologies [30], and in human robot interaction [31,32]. However, care must be taken when interpreting the results. There are many factors that can affect the measurements (e.g. ambient light, temperature, physical motion, electromagnetic noise), thus it is recommended that experimentation should be carried in a controlled environment. Also, it is recommended to measure more than one variable simultaneously in order to have a robust assessment.

Since psycho-physiological measurements haven’t been used in prosthetic applications, the main objective of this study was to explore the feasibility of using psycho-physiological measurements to assess cognitive effort when manipulating a robot hand with and without the usage of a sensory feedback system, and how these measurements are related to temporal and grasping performance when using the prosthetic hand in a static or fixed setting. In this way, we can examine the changes in different physiological variables and their relationship with the mental effort during the manipulation of a prosthetic hand, and how the usage of auditory information as the sensory feedback system affects their performance. The NASA TLX self-assessment questionnaire and the subject’s EEG, ECG, electro-dermal activity (EDA), and respiration rate were used to assess the cognitive effort.

## Methodology

### Robot hand

**Figure 1:**
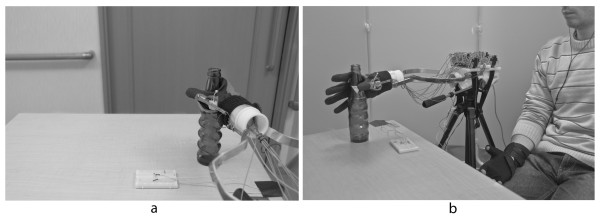
**Experiment setting. a**) Subject’s perspective of the robot hand during the experiment. The task was to grasp a bottle with the prosthetic hand. **b**) View of the whole experiment setting. The subject used a Data Glove to control the robot hand motion and bending sensors in the robot hand fingers were used to produce the auditory feedback cues.

A tendon driven robot hand was mounted on a tripod ready to grasp a bottle on a table, as shown in Figure 1. This type of robot hand has the advantage that the shape of the grip can adapt to almost any object shape passively without controlling many degrees of freedom. A Data Glove (5DT Ultra 5) was used to measure joint angles of the subject’s hand to manipulate the prosthetic hand. Since this Data Glove has only 1 sensor per finger, the controllability of the robot hand was reduced to 1 degree of freedom per finger. By limiting the degrees of freedom of the robot hand the subject was forced to pay more attention to position of the robot hand’s fingers instead of the position of his hand since one position of the robot hand can be achieved by different position of the subject’s fingers. Furthermore, for this experiment, the position of only the robot hand’s thumb, pointer, and middle finger was sampled (at 40Hz) for a cylindrical grasp (grasp a cylindrical bottle.)

### Sensory feedback system

Our research group developed a sensory feedback system using an auditory display, which is used as a redundant source of both kinematic and somatosensory information in prosthetic applications. This system was designed to enhance motor-sensory performance and awareness during the manipulation of a robot hand [21,22]. In this system different types of grasp (e.g. palm grasping, pinching) are mapped to different types of sounds (e.g. a piano, or a violin). This way the subject is able to easily know whether the prosthetic hand is doing or not his intended motion. Also, the robot hand motion needed to achieve the desired grasp is divided in different hand configurations. These hand configurations are mapped directly to the pitch of the grasping type sound, allowing the subject to dynamically monitor the motion of the robot hand. Furthermore, the trajectory of the robot hand’s fingers is compared to the expected trajectory and if one or more fingers are not following the expected trajectory (e.g. due to a mechanical malfunction or an obstacle) an error signal is generated and conveyed as an Auditory Icon. Auditory icons refer to sounds designed to convey information of a discrete event by analogy to everyday sound, for example the sound made by deleting files on a computer [33,34]. Similarly to convey grip force, a different sound from the one being used during the reaching phase is presented to the subject. The developed system uses OpenAL API (Creative Labs) to playback the sounds.

**Figure 2:**
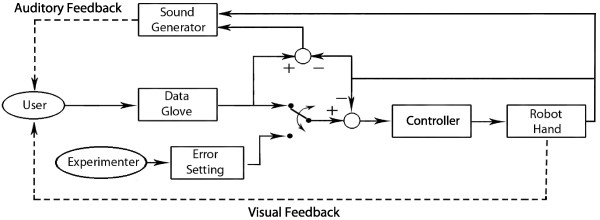
**Experiment setting block diagram.** The subjects controlled the robot hand’s motion using a Data Glove. The different profiles of the robot hand movements were mapped to sounds and conveyed back to the subjects. During tests, the experimenter forced one of the robot hand’s fingers to stop moving, which generated an error signal that was conveyed back to the subject as an auditory icon.

Figure 2 shows the block diagram of the system used to generate the auditory information. For this study only the cylindrical or palmar grasp was used during experimentation. The robot hand motion was divided in 8 different hand configurations and each configuration was mapped to 8 Piano major triads. The Hand Configuration 1 (C1) was considered to be the state when all the robot hand’s fingers were extended and was represented by a low C major triad. On the other hand, the hand configuration 8 (C8) denoted the state when all the fingers were completely flexed and was represented by a high C major triad. Since the task was to grasp a bottle, the robot hand never reached a completed flexed configuration (C8), therefore only the sound of 5 triads were presented to the subjects (C, D, E, F, G major triads). Finally, due to lack of pressure sensors in the robot hand used for this experiment, to indicate that the bottle was completely grasped a discrete signal was presented as another auditory icon. The fully grasp signal was triggered when the robot hand reached the hand configuration C6. C6 was realized when the angle of the subject’s finger was approximately 60.

### Experiment setting

10 male subjects, between 22 and 30 years old, right handed, and with no sensory or motor impairment participated in this study. They were asked to come for 2 consecutive days. On the first day the experiment objective, tasks, and experiment setting were explained. After they completed a 30 minutes guided training. On the second day each subject was tested in 3 different modalities: Auditory Feedback only control (AF), Visual Feedback only control (VF), and Audiovisual Feedback control (AVF). For each modality they were asked to perform 10 trials. At the end of each test, the subject had to answer the NASA TLX questionnaire. The order of the modality tests was randomly chosen for each subject.

The subjects were asked to wear the Data Glove and sit beside the prosthetic device so that the device is on the pose and position from which the subject could begin reaching and grasping motions with his own upper limb (Figure 1a). The subject’s perspective of this setting is shown in Figure 1b. Since the psycho-physiological variables (EEG, ECG, EDA, Respiration) were being recorded during the tests, they were told to move as little as possible during the trials. Although the subject was able to manipulate all 5 fingers, they were told that only the Thumb, Pointer, and Middle finger were going to be tested.

The experiment tasks consisted of closing the robot hand until the bottle was securely grasped (C6), which was achieved at approximately 60% of finger flexure as measured by the Data Glove, and then opening it again until the fingers were completely extended (C1). We emphasized on the fact that since the fingers controllability is only 1 degree of freedom, they cannot rely on the position of their own hand to monitor the robot hand motion because the robot hand mechanism will not always yield the same position as their own hand. The experimenter was able to induce errors randomly in the motion of the robot hand’s thumb, pointer, or middle finger during the closing or opening motion. Therefore, if an error was induced on the pointer, this finger was forced to stop moving. This will prevent the pointer to follow the expected trajectory, thus generating an error. Once the subject detected this error, he was required to stop his hand motion, check which finger generated the error (relying on visual only, auditory only, or audiovisual information), and move his respective finger backwards until the robot hand’s finger moved 1 position backwards as well. After this, continue with the motion. In this study, only 1 error per trial was presented.

The subjects were also required to fully grasp (exert enough force on the bottle so it would not slip if lifted) the bottle in order to finish the trial. A fully grasped bottle was indicated by a discrete auditory signal in the AF and AVF modalities. However in the VF modality the subject was required to approximate the grasp just by looking at the robot hand. If the bottle wasn’t completely grasped after finishing the open-close-open motion, the subject was required to close the robot hand again until the bottled was fully grasped and then open it again to finish the trial.

#### Auditory Feedback only (AF)

When testing this feedback modality the subjects’ eyes were covered, thus they had to rely only on the sounds to monitor the Robot Hand’s finger position. To start a trial the subject was asked to leave their own hand completely open and wait for the sound corresponding to C1 to be presented, then, start closing the hand until they heard the auditory icon that represented a complete grasp, and then open the hand until they heard the C1 sound again. They were required to repeat this for 10 times in a self-paced movement. If they detected an error happened they had to stop the motion, move the affected finger backwards until they heard the previous hand configuration sound, and then continue with the motion.

#### Visual Feedback only (VF)

For the VF modality, the subject was asked to monitor the robot hand’s finger motion only by looking at it. This is the same way by which current prosthetic hands have to be monitored and manipulated, thus can be regarded as a control group. A green LED was used to indicate when to start and finish each trial. The subject was asked to open his hand completely and wait for the LED to turn on, then, start closing the robot hand until the bottle was fully grasped. After that, the subject was asked to open the robot hand until the LED turned off. If the LED didn’t turn off, then the bottle was not completely grasped, or the error wasn’t detected and fixed, thus the subject had to close and open the robot hand again until the LED turned off.

#### Audiovisual Feedback (AVF)

In this modality the subject could monitor the robot hand using both the auditory and visual feedback as explained in the previous subsections.

### Performance Evaluation

We recorded the time taken to complete each trial and for each error to be detected and fixed. An error was considered detected and fixed when the subject moved the affected finger backward one position. This way we determined how long it took the subject to detect the error. We expected that, for the VF modality, the subjects were going to take more time to detect an error since it’s more difficult to notice when a finger stops moving, and also we expected the trials to last longer. Additionally, due to the lack of pressure sensors in the robot hand, a complete grasp was indicated by a digital signal. This is why, to assess the grasping performance, we measured how much the subjects flexed their fingers in order to achieve a complete grasp of the bottle with the robot hand. The output of the Data Glove was obtained as the percentage of the finger flexure, where 0% indicated a totally extended finger and 100% totally flexed finger. For this application, in order to achieve a complete grasp of the bottle the subject had to flex his fingers around 60%. For the VF modality we expected the subjects to flex their fingers more than in the other 2 modalities since they have to approximate visually when the bottle was completely grasped.

### Cognitive effort evaluation

We recorded several psycho-physiological variables (EEG, ECG, EDA, Respiration) during the different experimental modalities, and asked the subjects to fill the NASA TLX self-assessment questionnaire after each modality was finished. Baselines were recorded for 1 minute before the start of each test, during this time the subjects were asked to remain relaxed. As described before, for the AF modality the subjects had their eyes covered and they were asked to close their eyes. The BIOPAC MP30 was used to record all the psycho-physiological variables at a sampling rate of 1000Hz and the data was analyzed offline using Matlab 7.0. Also, due to the contamination of the psycho-physiological signals, only the data from 9 subjects could be analyzed.

#### Self-Assessment questionnaire

The NASA TLX was used to measure the subjective mental workload of the system. The overall workload score obtained is based on a weighted average rating of 6 sub scales: Mental Demands, Physical Demands, Temporal Demands, Own Performance, Effort, and Frustration. This scale has been successfully used to assess workload in different human-machine interfaces applications as described in [23,24,28]. In order to calculate the overall score each sub scale has to be weighted by presenting a pair of factors and asking the subjects to choose what they think contributed more to the workload of the task (there are 15 pair-wise comparisons). The final weights are obtained by summing the times each factor was selected. These weights range from 0 (no important) to 5 (more important than any other factors). After that, the subjects have to rate each of the factors in a scale divided into 20 equal intervals anchored by a bipolar descriptor (e.g. High/Low). Finally, each rating was multiplied by the weight given to that factor and all the results were summed and divided by 15.

#### EEG measurements

For this study, EEG measurements were carried out by 1 bipolar channel placed, according to the 10-20 standard in P4 (positive lead), T6 (negative lead), and A2 (ground lead), on the right side of the head. The position of the electrodes was chosen as recommended by the BIOPAC manual in order to reduce the blinking artifacts. The raw data was filtered with a 40Hz low pass filter. A manual inspection of the data was done in order to remove artifacts due to blinking. After that, the spectral powers of each trial were obtained for the Alpha (8 13Hz) and Beta (14 35Hz) bands. A Hamming window and the Fast Fourier Transform (FFT) were used to calculate the spectral powers of each frequency band [35,36]. Finally, the values obtained were divided by the baseline value of each modality in order to obtain an index of change of the signals [26] from the resting states.

#### Heart rate measurements

In this experiment, the heart rate measurements were carried out by a 3 lead configuration setting, that is, the Ground lead attached under the right clavicle, the Negative lead attached under the left clavicle, and the Positive lead attached to the lower left ribcage. The raw data was filtered with a 60Hz low pass filter, the number of spikes during each trial was recorded, and the inter-beat interval (IBI) between 2R peaks was recorded as well [37]. The approximate heart rate of each trial was calculated in beat per minutes. For the HRV, the FFT of the detrended IBI data set of the duration of each modality was obtained and the 0.1Hz component was extracted as described in [37]. After that, the HR and the HRV values were divided by each modality’s baseline in order to obtain a change index from the resting period.

#### Electro-dermal activity (EDA) measurements

In this study, the Skin Conductance Level (SCL) and the Skin Conductance Reactions (SCR) were obtained from 2 electrodes (Biopac SS57L) attached to the left hand’s index and middle finger. A baseline was obtained 2 seconds before the beginning of each trial and then the mean SCL of each trial was obtained. The final score for each trial was obtained by diving the trial’s level with the 2s baseline level. The SCR was recorded when the maximum level of a trial was higher than the mean of the 2s baseline, thus only one reaction was taken in account per trial. Due to a problem with one transducer during experimentation the data of only 7 people were taken in account to calculate the results.

#### Respiration rate measurements

Respiration rate was measured with Biopac’s SS5LB transducer, which measures the change in thoracic circumference. The raw data was band passed filtered between 0.05Hz and 1Hz, and the respiration rate was calculated as the amount of positive crest during each trial. After, the resulting values were divided by the baseline in order to obtain a change index from resting period.

## Results

The results obtained were analyzed in SPSS 16.0 using a Repeated Measures Analysis Of Variance (ANOVA) and the Greenhouse-Geisser correction estimates were used to measure the statistical effect. Table 1 shows a summary of the results from all psycho-physiological measurements and Table 2 shows the summary of the results when Subject 1 and subject 8 were not taken in consideration.

**Table 1:** Summary of psycho-physiological measurements results

	**Visual**	**Auditory**	**AudioVisual**	**Description**
	**feedback (VF)**	**feedback (VF)**	**feedback (VF)**	
NASA TLX	AF < VF	AF > AVF	VF > AVF	Higher perceived workload in VF modality followed very closely by the AF modality
Alpha Band	AF > VF^*^	AF > AVF	VF < AVF	Higher attentional demand in VF modality
Beta Band	AF < VF	AF < AVF	VF > AVF	Similar cognitive demand in all modalities.
HR	AF < VF	AF > AVF	VF > AVF^**^	Significantly higher Task difficulty and attentional demand in VF modality
HRV	AF > VF	AF > AVF	VF < AVF	No significant difference were found between modalities
SCL	AF < VF	AF < AVF	VF < AVF	No significant arousal during the tests
SCR	AF < VF	AF < AVF	VF < AVF	No significant arousal during the tests
Respiration Rate	AF > VF^**^	AF > AVF^**^	VF < AVF	Couldn’t be related to mental effort

^*^significance at p < 0.05.

^**^significance at p < 0.001.

**Table 2:** Summary of psycho-physiological measurements results without outliers (Subject 1 and Subject 8)

	**Visual**	**Auditory**	**AudioVisual**	**Description**
	**feedback (VF)**	**feedback (VF)**	**feedback (VF)**	
NASA TLX	AF < VF	AF > AVF	VF >AVF^**^	Significantly higher perceived workload in VF modality followed very closely by the AF modality
Alpha Band	AF > VF^**^	AF > AVF^**^	VF < AVF^*^	Significantly higher attentional demand in VF modality
Beta Band	AF > VF	AF < AVF	VF < AVF	Similar cognitive demand in all modalities.
HR	AF < VF	AF > AVF^*^	VF > AVF^*^	Significant Higher Task difficulty and attentional demand in VF modality
HRV	AF > VF	AF > VF	VF < AVF	No significant difference were found between modalities
SCL	AF < VF	AF < VF	VF < AVF	No significant arousal during the tests
SCR	AF < VF	AF < VF	VF < AVF	No significant arousal during the tests
Respiration Rate	AF > VF^**^	AF > AVF^**^	VF < AVF	Couldn’t be related to mental effort

^*^significance at p < 0.05.

^**^significance at p < 0.001.

### Performance

**Figure 3:**
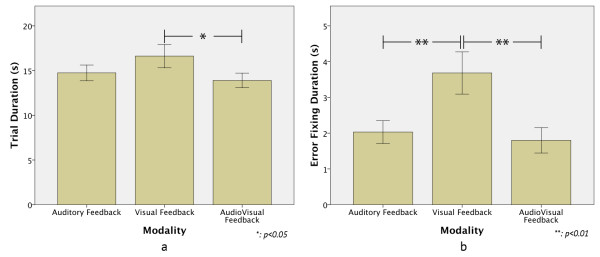
**Temporal performance results. a**) Trial Duration. It can be noted that the trial duration was lower when the auditory and the visual feedback was used simultaneously. **b**) Duration to detect and fix an error. An error was detected and fixed faster when the auditory display was used.

Figure 3a shows the mean of a trial’s duration of each modality for all subjects. A significant effect was found between modalities, F(1.4, 105) = 4.947 p < 0.05, and a Bonferroni Post Hoc test revealed that the trial duration of the VF modality was significantly longer than that of the AVF modality (p = 0.023), but there is no significant difference between the trial durations of the VF and AF modalities (p = 0.22). Figure 3b shows how long it took the subjects to detect and fix an error, and applying the same statistical test a significant difference between modalities, F(1.2,48) = 14.93 p < 0.01, was found. Post Hoc test showed no significant difference between the AF and AVF (p = 0.478), but there was a significant difference between the modalities AF and VF (p < 0.001) and between the AVF and VF (p < 0.001). These results were expected because during the VF modality it is more difficult to detect an error fast.

**Figure 4:**
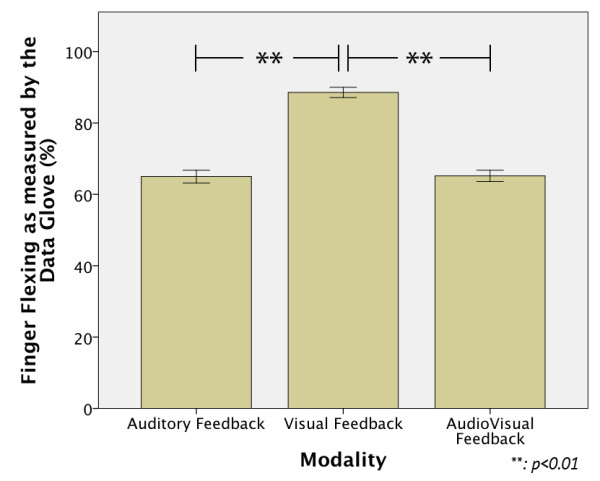
**Finger flexure results.** The finger flexure was measured as a percentage value where 0% indicated a fully open hand pose and 100% indicated a fully closed hand pose (e.g. fist). From the results it can be noted that the Auditory feedback allowed the subject to know more accurately when the bottle was completely grasped.

Figure 4 shows how much the finger flexed his finger when grasping the bottle as measured by the Data Glove, where 0% refers to the fingers completely extended and 100% to the fingers completely flexed. A statistical difference was found between modalities, F(1.9,135) = 184.99, p < 0.01. The Post Hoc tests showed that there was no significant difference between AF and the AVF modalities (p = 0.48), but a significant difference between AF and VF (p = 0.001) and AVF and VF (p = 0.001) was found. These results were also expected because during the VF modality the subjects have to rely on what they see to achieve a complete grasp.

### Nasa TLX results

**Figure 5:**
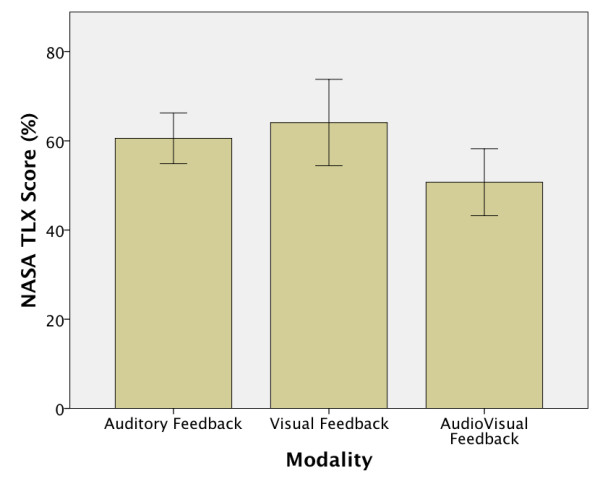
**NASA TLX scores.** The NASA TLX mean scores showed that, in general, the VF modality was ranked with the highest mental workload, followed very closely by the AF modality when manipulating the prosthetic device.

**Figure 6:**
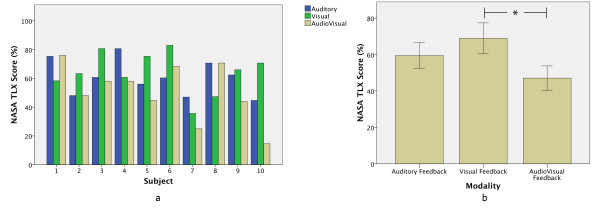
**NASA TLX scores without outliers. a**) Subject 1 and Subject 8 were the only ones that reported that the mental effort in the VF modality was lower than in the other 2 modalities, which were considered to be similarly difficult. **b**) Considering Subject 1 and Subject 8 as outliers, the results showed the same tendency, but with a significant effect between modalities.

The NASA TLX mean scores showed that, in general, the VF modality was ranked with the highest mental workload, followed very closely by the AF modality when manipulating the prosthetic device (Figure 5). On the other hand it seems, from the overall results, that the subjects agreed that the mental workload is lower when a multimodal feedback (AVF modality) is used, although no statistical difference was found between modalities, mainly due to the variability between the subjects’ ratings. Comparing the subject’s individual scores (Figure 6a) it is possible to notice that Subject 1 and Subject 8 were the only ones that reported that the mental effort in the VF modality was lower than in the other 2 modalities, which were considered to be similarly difficult. Therefore, if we consider Subject 1 and Subject 8 as outliers, and exclude their scores from the statistical analysis, the results showed the same tendency, but with a significant effect between modalities F(1.8,12.9) = 7.954 p = 0.006 with a significant difference (p < 0.05) found between the VF and the AVF modalities (Figure 6b).

### EEG results

**Figure 7:**
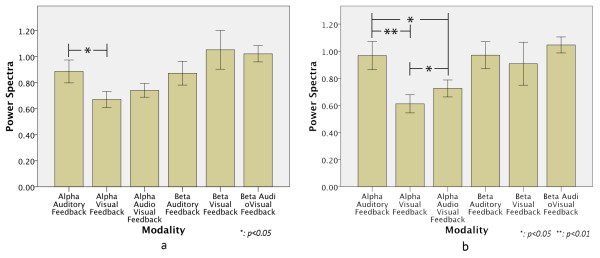
**EEG results. a**) The EEG mean power spectra of the Alpha and Beta band of each modality for all subjects. A higher Alpha suppression was found for the VF modality. **b**) When considering Subject 1 and 8 as outliers, we found a significantly statistical effect between the modalities. These results points out a higher attentional demand in the VF modality.

The EEG mean power spectra of the Alpha and Beta band of each modality for all subjects are shown in Figure 7a. We observed that there was a higher Alpha suppression for the VF modality compared to the other 2 modalities (F(1.4,105)=6.455, p=0.006). Comparing the VF modality with the AF modality, a significant difference of p=0.013 was found, whereas comparing the VF modality with the AVF modality, no significant difference was found. The AF and AVF modalities didn’t show any significant difference either. The Beta band didn’t show any significant effect between modalities.

As expected from this kind of measurements a large variability between subjects was found. The results pointed out that Subjects 1 and 8 also had a different tendency than the rest of the subjects. Therefore, by treating Subjects 1 and 8 as outliers we found a significantly statistical effect between the modalities (F(1.27,75.2) = 14.06 p < 0.001) and the Post Hoc test showed a significant difference between all modalities: p < 0.01 between AF and VF, p = 0.011 between AF and AVF, and p = 0.006 between VF and AVF. Furthermore, for the Beta band no significant differences were obtained between modalities. This result can be observed in Figure 7b.

### ECG results

**Figure 8:**
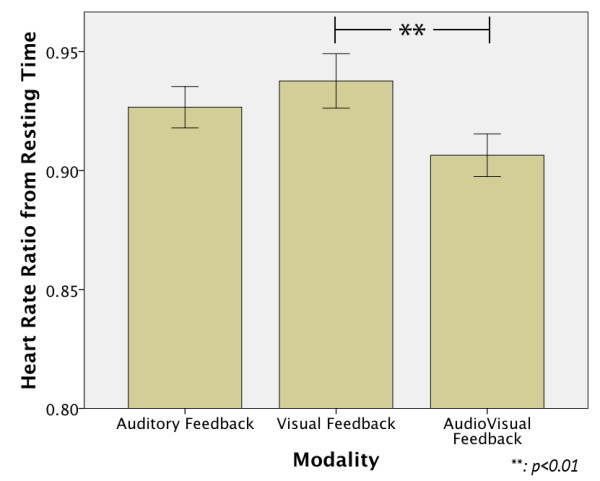
**ECG results.** The VF modality showed the lowest deceleration of all the groups, followed very closely by the AF modality, while the AVF showed the highest deceleration. Which points out a lower mental effort and more focused attention in the AVF modality.

The Heart Rate results are shown in Figure 8. It can be noted that for all modalities there was a deceleration of the heart rate from resting values, and the statistical test showed a significant effect between modalities F(1.7,133.6) = 6.944 p = 0.002. This effect was due to a significant difference between the VF and the AVF modalities (p = 0.004), since the VF modality showed the lowest deceleration of all the groups, followed very closely by the AF modality, while the AVF showed the highest deceleration. Additionally, the Heart Rate Variability results showed the lowest variability for the VF modality, but no statistical effect was found.

Considering Subject 1 and 8 as outliers for the other measurements, the AVF modality showed the highest deceleration, but a smaller statistical effect between modalities was found (F(1.9,111.56) = 3.69 p = 0.03). The HRV didn’t show any statistical difference between modalities.

### Respiration rate and electrodermal activity results

The respiration rate increased from resting values for all feedback modalities and was significantly higher for the AF modality when compared with the VF (p < 0.001) and with the AVF (p = 0.003) modalities, and the respiration rate for the VF and the AVF modalities was very similar. On the other hand, the electro-dermal activity (EDA) didn’t show any statistical effect between modalities for all subjects, which points out that the subjects were not aroused during the experiments.

### Scores correlations

Finally, the correlations between the performance and the variables used to measure mental effort are shown in Table 3. We can see a strong relationship between the subjective ratings and the performance results. Also a significant correlation between the performance and some of the psycho-physiological variables was found.

**Table 3:** Spearman correlations between performance results and the psycho-physiological measurements

	**Alpha**	**Beta**	**HR**	**HRV**	**SCL**	**SCR**	**Resp**	**NASA TLX**	
Duration	0.029	0.109	0.191^**^	0.348^*^	-.376^**^	-0.193	0.220^**^	0.511^**^	
Detecting	-0.100	-0.036	0.242^**^	0.102	-.215^**^	- 0.104	0.136	0.376^*^	
Grasping	- 0.102	0.116^*^	0.041	-0.318	-0.107	-0.256	-0.098	0.436^**^	

^*^significance at p < 0.05.

^**^significance at p < 0.001.

## Discussion

### Performance

The performance results presented in this paper showed a better temporal performance in the AF and AVF modalities than in the VF modality for detecting and correcting an error in the robot hand’s finger trajectories. This is an expected result for the experiment setting used in this study because it is well known that auditory cues convey faster and more accurately the moment an error occurred, than when the subject is only looking at the hand [33,34,38-40]. Also, it has been considered in literature that auditory cues enhance visual perception when the sound is congruent with visual feedback [33,34,38,40-43], which explains why the performance in the AVF modality was the best for all subjects. Another problem of relying only on vision to manipulate a robot hand is that while reaching and grasping there can be blind spots that prevent the user from successfully monitor the motions. For example, in this experiment the bottle partially blocked the pointer, middle, ring and little finger, making the detection of errors in these fingers more difficult for the subjects (Figure 3). Similar results were obtained by S. Kousidou et al. [38], C. Ghez et al. [39], and H. Huang et al. [41]. They showed that subjects performed smoother motions in less time when auditory feedback was used for rehabilitation therapies.

Also, since the subject had to approximate the grasping force of the bottle in the VF modality, we were expecting that in some trials the subject could not grasp completely the object, thus, had to close and open the hand again. This situation happened approximately once per subject because most of the subjects opted to flex more than necessary their fingers, which can be seen from the grasping finger flexion results presented in Figure 4. These results showed the low ability of the VF modality to approximate correctly the grasping force necessary to be applied to an object, therefore, the user will end up doing more mental and physical effort. Richard P. et al. showed similar results for force feedback when manipulating virtual deformable objects [44]. To make this comparison more realistic, force sensors will be added to the robotic hand in order to investigate this situation directly. In that way, the subject will be required to approximate the grasping force as well, but using sounds.

### ECG measurements

Heart rate is the most used physiological variable to assess mental workload since is the easiest to measure and calculate [25]. In general, larger heart rate deceleration has been associated with a decrease in the difficulty level of a task [27,45], and it is important to take in consideration that these changes are related to the type of task used in the experiments. Kramer, A. [29] discussed that Heart Rate deceleration have been related to intake of information (e.g. visual or auditory detection) rather than rejection of information (e.g. mental arithmetic, memory retrieval), and Pascalis, V.D. et al. [45] argued that heart rate deceleration is an expression of stimulus processing and a focus in attention priorities. Our results are in agreement with these findings since the subjects used visual and auditory information to achieve the task and deceleration was found for all modalities. Also, we can assume that the larger deceleration found in the AVF might be related to a higher attentional state and consequently lower mental effort was required in the task, as described in [27,29,45,46], because the sensory integration of auditory and visual feedback helps the subjects to focus more in the task at hand [47]. Also, this attentional state can be attributed to the enhancing factor of the visual information by the auditory cues, as discussed in [40]. This can also be observed from the heart rate results obtained in the AF modality because during the AF modality some subjects showed a higher deceleration while others showed lower deceleration in the heart rate. This shows that individual difficulties play a big role in processing or giving meaning to the auditory cues, which seems to disappear when the visual input is also available (AVF modality). Also, the low HR deceleration showed in the VF modality indicates a higher difficulty in the task, and can be attributed to a higher attentional demand state as discussed in [25,27,29]. It is possible that in this modality the subjects were more distracted from the task (e.g. occasionally looking to another point in the room).

### EEG measurements

The EEG has been widely used for assessing cognitive effort and attentional demand because it’s a direct link to activity in the CNS and, when compared to fMRI, relatively easy to setup. Studies have found a sensitivity of the EEG to mental effort and attentional demands. For example, in [26] the results showed a higher alpha suppression for increased task difficulty in a simultaneous dual task of tracking and mental arithmetic. Ray, W. et al. [48] compared the effect of attentional demand (intake-rejection of sensory information) on the EEG during cognitive and emotional tasks. They found that increases in Beta band activity are more related to cognitive (rejection of sensory information) and emotional processes, and that a reduction in Alpha band activity reflects attentional demand to sensory information processing (intake). Also, Shier, M. A. [49] showed how simulated driving imposes significant demands on attention by a higher suppression of the alpha band.

Furthermore, Fournier, L. et al. [50] and Kramer, A. [29] point out that Alpha band decreases with increase of task difficulty when external information processing increases. Therefore, the small differences obtained in the Beta band and the suppression of the Alpha activity for all modalities point out that the effect of manipulating the robot hand increased the subject’s attentional demand, but didn’t show a significant effect in cognitive processing (e.g. thinking about the meaning of the sounds or processing which finger had an error). Therefore, the EEG results obtained in this study can be related more to changes in attentional demand than changes in cognitive effort since the changes in the Beta band were not significant.

Alpha activity is suppressed with visual stimulation because it increases cortical activation [51], and that is the reason why the alpha activity increases when a person closes his eyes. This is why we decided to calculate the change ratio from resting values of the EEG data in order to eliminate this bias, but nevertheless the results showed a large difference between the AF modality and the other 2 modalities. From this we can argue that, in our experiment, when the subjects’ eyes were covered visual processing was cut away, reducing external distractions, thus decreasing cortical activation greatly. On the other hand, when their eyes were not covered, the subjects’ brain was not only processing the robot hand activity, but also all the things around it, which increased the attentional demand. It is because of this point why it is difficult to compare the Alpha band results between the AF modality with the AVF and VF modality. Nevertheless, when using the auditory information along with vision (AVF modality) there was considerably less alpha suppression than when only relying on vision (VF modality). This can be attributed to an enhance or “pop-out” effect on the visual input by the auditory information, as discussed in [40] and [47]. This allowed the subjects to pay more attention to the robot hand’s motions. Also, as discussed in [43], it is possible that sensory integration of visual and auditory input helped the subjects to understand better and faster what was happening during the task, decreasing the attentional demand, thus improving mental processing and improving their performance.

We wanted to explore the possibility to measure workload using only 1 EEG channel to reduce the subject’s discomfort of attaching many sensors for future experiments. Although our results and the results obtained by Huang et al. [36] showed that it is possible to measure to a certain extent the mental workload using 1 EEG channel, it is better to use at least 2 to 4 channels to have a better resolution, allowing more localized observation and better resolution of the brain activity.

### Respiration rate and electro-dermal activity

Respiration rate, in [50,52], was found to be significantly faster during a tracking and mental arithmetic multi-task condition, i.e. more demanding tasks. In this study, the results presented didn’t show any significant effect on task difficulty between VF and AVF, but in the AF modality the subjects had a significant increase in the respiration rate. In [30] and [32] it is argued that increases in respiration rate are directly related to increases in arousal, which might be related to having the eyes covered, but the EDA obtained showed a very low arousal state during all modalities. However, the task used in this study might have been too simple to elicit a strong EDA activity. On the other hand, as indicated by Gomez et al. [53], the speed in which the sounds changed could have affected directly the respiration rate, which can explain why in the AF and the AVF modalities the subjects had a larger respiration rate.

### General discussion

In this preliminary study we used the NASA TLX and different psycho-physiological measurements to explore the subject’s mental effort when manipulating a robot hand. According to what has been claimed or assumed in literature, we were expecting that the needed mental effort to manipulate the robot hand would be similar between the VF and the AF modality (unimodal feedback) and to be significantly lower during the AVF modality (multimodal feedback). The results obtained from the NASA TLX, the ECG and the EEG points out that the difficulty of the task was considerable reduced when using a multimodal feedback scheme (during the AVF feedback). However, for this study, the changes in mental effort and performance can be associated to changes in the subject’s attentional demand rather than cognitive effort, as has been regarded in literature.

In general, the VF modality was rated to be the most mentally demanding task, which relates to be the modality with the highest suppression in the Alpha Band, the lowest deceleration in HR and the lowest performance results. As discussed before, all of these results can be attributed to an increase of attentional demand since the subjects need to be looking at the robot hand at all times in order to accomplish the task. Also, the NASA TLX and the ECG results were similar during the AF and VF modality thus can be also attributed to an increase of attentional demand although the performance was better during the AF modality. The performance results during the AF modality were expected since auditory cues allowed the subjects to detect and react faster to the errors. During this modality, having the eyes covered could have influence the results obtained for the EEG. On the other hand, the results showed that during the AVF modality the attentional demand was reduced significantly (low Alpha suppression, high HR deceleration) and was rated to be the less mentally demanding modality in the NASA TLX and can be attributed to the “pop out” or enhance effect due to the sensory feedback system [40,43,44].

Furthermore, another important factor showed by the results is how individual differences could affect the perception of a sensory feedback system. In this study Subject 1 and 8 considered the VF to be the less mentally demanding task in the NASA TLX as opposed to the other subjects and when removing their data from the analysis we found out a significant variation on the EEG and ECG results. After the experiments these 2 subjects commented that they had difficulties remembering the meaning of the sounds and that were a little overwhelmed by the auditory feedback during the trials. However, they also showed better performance in the AVF modality, which could be an indication that the multimodal sensory feedback helped them improve the performance, but increased their mental demand and could have an important effect when using the system for longer periods of time. This is a very important point that needs to be researched further. Table 4 shows a summary of the insights obtained in this study concerning the psycho-physiological assessment of mental effort, compared with the claims in other studies.

**Table 4:** Comparison of the psycho-physiological assessment of mental effort between this study and other studies in literature

				
	**Attention**	**Mental effort**	**Attention**	**Mental effort**
Alpha Activity	++	+	++	+
Beta Activity		+		++
HR Deceleration	++	++	++	++
HRV		+		++
SCL	-	-		+
SCR	-	-		+
Resp.	-	-		+

^++^Very useful.

^+^useful.

^-^inconclusive.

**Figure 9:**
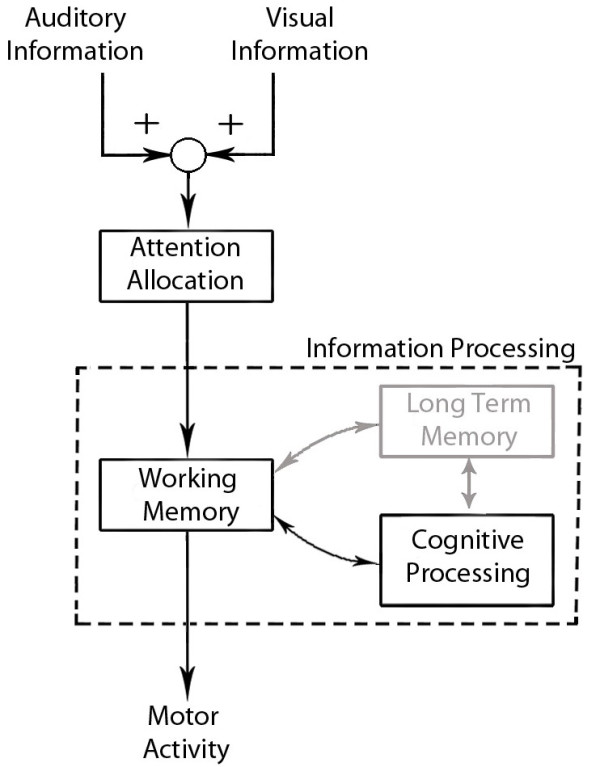
**Attentional model.** Sensory information is integrated, enhancing or reducing the attention to a specific task. Then, this information is processed in order to understand the meaning of the sensory input and to decide what actions should be taken. Long term memory wasn’t taken in consideration in this study.

The results obtained in this study suggest that sensory integration plays an important role for successfully manipulating the robotic hand from a point of view of attention to the task and performance, regardless of individual differences. Although the results obtained are preliminary, they can be interpreted by the model illustrated in Figure 9, based on the famous attention model described by Treisman, A. et al. [54] and Deutsch, J. et al. [55]. The high performance and low mental effort showed in the AVF modality can be described by an early integration of the auditory and visual information, which enhance the perception of the task. This enhancement helps to allocate attention to different situations, allowing the subject to process faster the important information. As showed and discussed in [40,43,47], if the sensory information is coming from the same source (e.g. Robot Hand) and is congruent with one another the overall perception of the activity will be enhanced. On the other hand, if the information is contradictory or unrelated, the overall perception will be decreased (e.g. more distraction or attentional demand). Also, different studies in neuroscience have shown a co-activation by both visual and auditory information in different portions of the brain [56-58], which points out an integration of sensory information in an early stage of processing. Moreover, in [56] the authors discussed that attention is allocated to ensure that the appropriate task relevant information is passed on to decision making and behavioral control systems. Also, as discussed in [59], the multimodal feedback can have a beneficial effect on workload and performance. The performance showed in the VF and AF modality can be describe as well with the model in Figure 9. In the VF and AF modalities less information was available than in the AVF modality, thus the subjects needed to process the meaning of this information a little further (e.g. in which finger the error happened or when an error happened) [40,43,58].

Furthermore, it seems that the task used in this study didn’t have a strong impact on the mental workload since it was a static setting. Therefore, the subjects didn’t have to spend too much effort to think about what is the meaning of a sound, or how to react if an error happened. Also, since the training session was held a day before the trials, and a short-term remainder of the sounds was held right before the trials, the long-term memory access and its effect on performance and the psychophysiological variables weren’t explored.

## Conclusion

In this preliminary study we explored the feasibility of using psychophysiological measurements to assess mental effort directly when manipulating a robot hand with and without the usage of a sensory feedback system. For the task used in this study the results point out that the use of an auditory display as a sensory feedback system reduces the attentional demand needed to complete the task, rather than the cognitive effort as it is considered in literature. Therefore, performance improvements when using auditory cues, along with vision (multimodal feedback), can be attributed to a reduced attentional demand during the task, which can be attributed to a visual “pop-out” or enhance effect. It will be very important to investigate whether this result can be extrapolated to other sensory feedback schemes as well. Furthermore, the psycho-physiological and self-assessment questionnaire results showed how individual differences could affect the perception of a sensory feedback system, affecting the results greatly.

From the results we consider that the NASA TLX, the EEG’s Alpha and Beta band, and the Heart Rate could be used to further evaluate sensory feedback systems in prosthetic application. However, it is important to design a new experiment where the subjects have to use the system for longer periods of time in order to take into account also the fatigue effect. Also the experiment should be design for daily living situation where a dynamical manipulation of the prosthetic hand is needed, thus the complexity of the task is larger. Moreover, we should explore the emotional aspect of using a robot hand in order to assess anxiety and engagement. In this way we can further corroborate, validate and extend the model presented in this study.

## Competing interests

The authors declare that they have no competing interests.

## Author’s contributions

JG: carried out the design, development, execution and analysis of the experiments. Also, he was in charge of drafting the paper. HS: participated in the development and execution of the experiments. MS: participated in the development of the material needed for the experiments. WY: participated in the design and analysis of the experiments. Also, he participated in drafting the paper. All authors read and approved the final manuscript.
